# Metastasis of Laryngeal Squamous Cell Carcinoma to Bilateral Thigh Muscles

**DOI:** 10.1155/2014/424568

**Published:** 2014-12-14

**Authors:** Zarah Lucas, Akash Mukherjee, Stanley Chia, Irina Veytsman

**Affiliations:** ^1^Division of Hematology and Oncology, Washington Cancer Institute, Washington, DC 20010, USA; ^2^MedStar Washington Hospital Center, 110 Irving Street, Washington, DC 20010, USA; ^3^Department of Otolaryngology, MedStar Georgetown University Hospital, Washington, DC 20007, USA

## Abstract

*Importance*. Laryngeal cancer infrequently results in distant metastases, but metastasis to skeletal muscle is extremely uncommon. *Observations*. A 55-year-old male presenting with progressive dyspnea and hoarseness was found to have Stage IVA T4aN2cM0 laryngeal cancer and eventually underwent total laryngectomy. Before the patient could be started on adjuvant chemoradiation, the patient developed masses on both thighs. Biopsy revealed metastatic squamous cell carcinoma consistent with the primary laryngeal cancer. He was offered palliative chemotherapy; however, he developed new soft tissue masses to the left of his stoma and in the prevertebral area one week later. He also had new cervical and supraclavicular nodes and a pathological compression fracture of L3. Patient died within 4 months of diagnosis. *Conclusions*. Distant metastasis such as skeletal metastasis portends a poor prognosis. Further studies are required to determine the best course of treatment in these patients.

## 1. Introduction

It is estimated that 12,630 men and women will be diagnosed with laryngeal cancer in 2014. Distant metastasis occurs in up to 19% of all cases [1]. The most common site of distant metastases from laryngeal cancer is the lung. Distant metastasis to the skeletal muscle is extremely unusual. To our knowledge, there are only two reported cases of metastatic laryngeal carcinoma to the musculature of the lower extremities in the literature [2]. We present the case of a patient with locally advanced laryngeal squamous cell carcinoma who developed skeletal muscle metastases to both thighs shortly after definitive surgical treatment of the primary cancer. This case raises awareness of skeletal muscle metastasis and its implications in the management of high-risk laryngeal cancer patients.

## 2. Case Presentation

**Figure 1:**
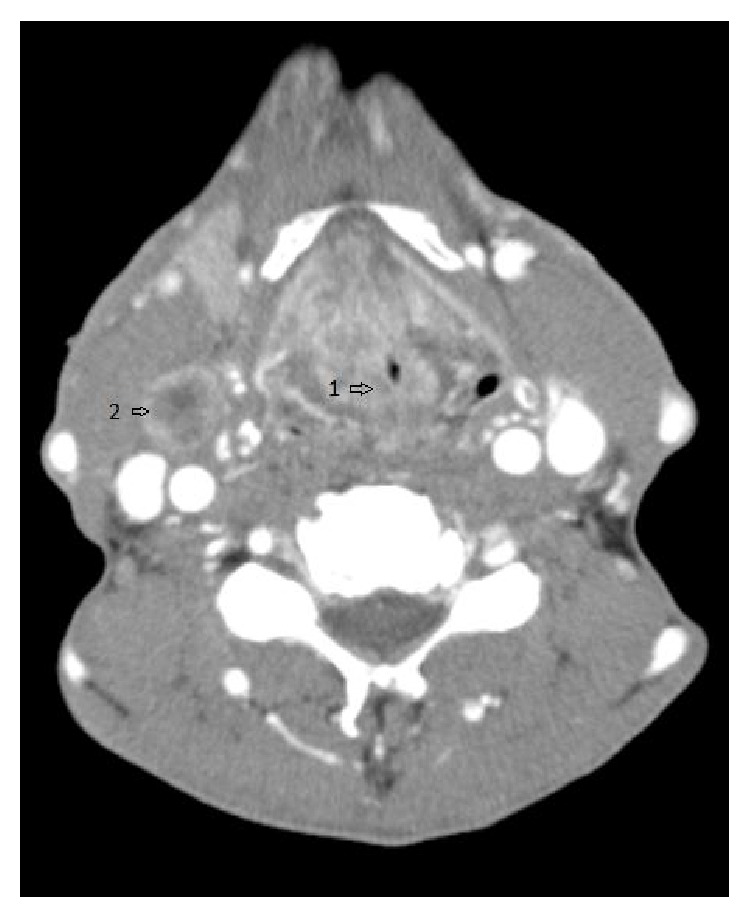
Supraglottic-level CT showing airway narrowed by glottis mass (1) and lymph node metastasis (2).

A 55-year-old African-American male with significant smoking and drinking history presented with progressive dyspnea and hoarseness over five months. He had stridor and multiple enlarged right lymph nodes (level II and level III) on exam. Flexible laryngoscopy revealed an obstructing mass of the glottis; however the vocal fold mobility could not be assessed. Computed tomography (CT) image of the neck revealed an extensive transglottic mass with significant laryngeal narrowing and cartilage invasion as well as multiple enlarged right neck and retropharyngeal lymph nodes (Figure 1). Urgent tracheostomy was performed due to airway compromise. Direct laryngoscopy revealed a transglottic mass that originated at the level of the vocal folds bilaterally, extending to the subglottic region approximately 1 cm past the true vocal cords. The inferior extent of the transglottic mass was difficult to discern. Multiple biopsies were performed and pathology revealed poorly differentiated squamous cell carcinoma. Chest CT imaging revealed no distant metastases.

After discussion of treatment options, the patient underwent total laryngectomy with bilateral neck dissections, cricopharyngeal myotomy, and primary tracheoesophageal puncture. Invasive, poorly differentiated squamous cell carcinoma was noted in the glottis and supraglottis with cartilage invasion. Metastases to bilateral cervical lymph nodes and right retropharyngeal lymph nodes with extracapsular extension were noted. Surgical margins were free of tumor. The final pathologic stage was determined to be Stage IVA (T4aN2cM0).

**Figure 2:**
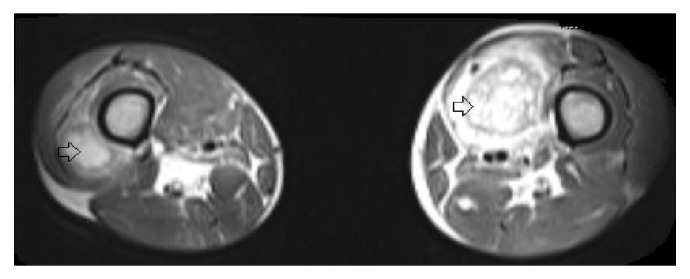
MRI of bilateral thighs showing contrast-enhancing metastatic skeletal lesions (arrows).

Due to the presence of high-risk features, including multiple cervical and retropharyngeal lymph nodes involved by the tumor with extracapsular extension, the plan was to proceed with adjuvant concurrent chemoradiation with cisplatin. However, his recovery was complicated by significant dysphagia, unsuccessful attempts at percutaneous endoscopic gastrostomy tube placement, 12 kg weight loss,* Escherichia coli* bacteremia, and multiple readmissions to the hospital that precluded conduction of positron emission tomography (PET)/CT scan. Less than two months after surgery, the patient reported pain in the left thigh and back. On examination, he was found to have bilateral, firm, nontender thigh masses, each measuring approximately 6 cm in maximal diameter with no associated erythema, warmth, or fluctuance. CT scan of the lower extremities showed a contrast-enhancing mass on the left vastus medialis measuring 4.5 cm × 4.5 cm × 7.7 cm and a lesion on the right vastus lateralis measuring 3.3 cm × 2.2 cm × 4.7 cm. Magnetic resonance imaging (MRI) was also performed to further evaluate the masses (Figure 2). Ultrasound-guided core biopsy revealed metastatic squamous cell carcinoma in both thigh masses.

Treatment options were discussed and the patient agreed to start palliative chemotherapy. However, he was readmitted one week later complaining of shortness of breath, increasing back pain, and new neck masses. A CT of the neck showed new soft tissue masses to the left of the patient's stoma, necrotic prevertebral soft tissue masses, and new cervical and supraclavicular nodes. MRI revealed a pathological compression fracture of L3 with posterior protrusion of bony cortex into the spinal canal. Due to the rapidly progressive course of the disease and poor performance status, the decision was made to proceed with palliative radiation to the spine only and he was placed under hospice care. He passed away within 4 months of the initial diagnosis of cancer.

## 3. Discussion

Laryngeal cancer metastases are most typically noted locoregionally to the cervical lymph nodes. Distant metastasis is seen much less frequently. The most commonly affected sites for distant metastases are the lungs (66%), bone (22%), liver (10%), mediastinum, and bone marrow. Distant skeletal muscle metastasis from laryngeal cancer is extremely rare [2]. To the best of our knowledge, this is only the third case of laryngeal cancer with metastases to skeletal muscles of the lower extremities reported in the English literature [2, 3]. Skeletal muscle metastases from laryngeal cancer to scapular muscles [4], internal obliques [5], and rectus abdominis [6] have been described.

Skeletal muscle metastases from any cancer are uncommon. In a review of 8,825 radiologic studies (CT, MRI, and PET/CT images) from one institution, 52 patients were found to have metastatic skeletal tumors. Twenty-one cases (40%) were from a lung cancer primary tumor. Other primary sites included the breast, rectum, pancreas, ovary, and larynx [5]. Skeletal muscle is thought to be resistant to invasion by cancer. Blood flow within the muscle is highly variable, especially during exercise. This makes tumor implantation in the endothelium quite difficult. Contraction of the muscles can also cause a biomechanical death of tumor cells. In addition, the production of lactic acid and protease inhibitors within the muscle hinders the growth of tumor cells by blocking enzyme-dependent tumor implantation [7].

Skeletal muscle metastases may be painful and palpable on presentation, as seen in our case, or the lesions could be asymptomatic. These tumors appear as enhancing lesions on CT imaging. The sensitivity of PET/CT imaging in detecting intramuscular metastases is greater than that of CT or PET alone. Unfortunately, PET/CT was not performed in our patient because his multiple inpatient admissions prevented him from scheduling the study. Thus, we cannot completely rule out an asymptomatic distant metastasis in our patient. However, there were no lesions noted in the extremities at the time of initial presentation or during the perioperative course. The extremity lesions were not apparent until 2 months after the surgery, at which time he was found to also have aggressive local recurrent disease and pathologic fracture of the spine, making a second primary in the extremities unlikely. Although distant metastasis is very uncommon on initial presentation in head and neck cancer, the diagnosis of Stage IVC disease through complete staging would have changed the management. Specifically, an extensive surgery would not have been done and palliative radiation to the neck could have been offered instead. This case exemplifies the importance of complete staging, including PET/CT.

On presentation, our patient had locally advanced laryngeal cancer (Stage IVA pT4aN2cM0). Despite complete excision of the primary tumor and cervical metastases, there were multiple features of the disease that portended a poor prognosis, including numerous bilateral cervical lymph node involvement, retropharyngeal lymph node involvement, and extracapsular extension [8, 9].

The limited number of cases of skeletal metastases from laryngeal carcinoma makes it difficult to determine the treatment of choice. The prognosis for such patients is poor. In the few selected cases with isolated muscular metastasis, particularly after a long disease-free interval, surgical excision can be pursued provided that locoregional control of the tumor has been achieved. In one case report of laryngeal carcinoma with isolated metastasis to the gluteus maximus and no evidence of any other metastatic lesion or locoregional recurrence, the patient underwent complete surgical excision of the lesion in the gluteus. On follow-up thirteen months later, there was no evidence of recurrence [3]. In a second case report of laryngeal carcinoma with isolated metastasis to the rectus femoris and no other evidence of metastasis, the patient also underwent complete surgical excision. The patient did well for nearly four years, at which time he died from brain and suprarenal metastases [2].

Currently, the recommended treatment for distant metastases from head and neck cancer is either enrollment into a clinical trial or systemic chemotherapy with cetuximab and platinum-based agents [10]. The EXTREME (Erbitux in First-Line Treatment of Recurrent or Metastatic Head & Neck Cancer) study involving recurrent or metastatic head and neck cancer demonstrated that the addition of cetuximab to platinum-based chemotherapy with 5-fluorouracil significantly prolongs the median overall survival, from 7.4 months to 10.1 months [11]. Treatment should always be individualized in such cases and, for patients with poor performance status and multiple comorbidities who would be unable to tolerate cetuximab and chemoradiotherapy treatment, comfort care and palliative measures should be offered to improve the quality of their life.
